# MRSA in pig farming: the emerging role of flies in antimicrobial resistance: a cross-sectional study

**DOI:** 10.1186/s40813-025-00459-0

**Published:** 2025-09-30

**Authors:** Flora Hamar, Igor Loncaric, Tanja Bernreiter-Hofer, Adriana Cabal Rosel, Anna Stöger, Monika Palle-Reisch, Werner Ruppitsch, Annemarie Kaesbohrer, Andrea Buzanich-Ladinig, Michael Bluemlinger, Lukas Schwarz

**Affiliations:** 1 Clinical Centre for Population Medicine in Fish, Pig and Poultry, Clinical Department for Farm Animals and Food System Science, University of Veterinary Medicine Vienna, Vienna, Austria; 2 Institute of Microbiology, Centre of Pathobiology, Department of Biological Sciences and Pathobiology, University of Veterinary Medicine Vienna, Vienna, Austria; 3 Centre for Food Science and Veterinary Public Health, Clinical Department for Farm Animals and Food System Science, University of Veterinary Medicine Vienna, Vienna, Austria; 4 Institute for Epidemiology and Infectious Disease Surveillance, Austrian Agency for Health and Food Safety (AGES), Vienna, Austria; 5 Institute of Medical Microbiology and Hygiene, Austrian Agency for Health and Food Safety (AGES), Vienna, Austria; 6 Institute of Hygiene and Medical Microbiology, Medical University Innsbruck, Innsbruck, Austria

**Keywords:** *Staphylococcus aureus*, MRSA, Livestock-associated MRSA, Flies, Antimicrobial resistance, Pig farms, Biosecurity, One health

## Abstract

**Background:**

The bacterium *Staphylococcus aureus*, particularly its methicillin-resistant form (MRSA), presents significant public health challenges due to its resistance to β-lactam antibiotics and frequent multidrug resistance. Livestock-associated MRSA (LA-MRSA), especially clonal complex 398, has become a concern in intensive pig farming, with emerging evidence suggesting flies may act as vectors. This study investigates the occurrence and molecular characterisation of MRSA in house flies (*Musca domestica*) and stable flies (*Stomoxys calcitrans*) on Austrian piglet-producing farms to understand their role in MRSA transmission and resistance dissemination.

**Results:**

MRSA was detected in 41.7% of 24 pig farms, with isolates identified in house flies (53.2%), stable flies (19.1%), boot sock samples (17.0%), and dust wipe samples (10.6%). All isolates were cefoxitin-resistant and belonged to CC398, carrying resistance genes such as *mecA*, *erm*(A), *erm*(B), *erm*(C), *lnu*(G), *lsa*(E), *vga*(E), *dfrG*, and in ciprofloxacin-resistant isolates, mutations in the quinolone resistance-determining regions (QRDRs) of the genes *gyrA* and *grlA* were observed. Beside resistance to β-lactams, resistance to tetracycline (100%), erythromycin (74%), clindamycin (74%), and ciprofloxacin (32%), trimethoprim-sulfamethoxazole (17%) was observed. Multidrug resistance (MDR) was observed in 94% of isolates. House flies (26%) were more frequently associated with MRSA carriage than stable flies (9.4%), indicating their potential as significant vectors. Environmental samples (boot sock and dust wipe samples) further confirmed widespread barn contamination.

**Conclusions:**

This study highlights the prevalence of LA-MRSA in Austrian pig farms and identifies flies as vectors contributing to its spread. These findings emphasise the importance of robust biosecurity measures, including effective fly control and stringent hygiene protocols, to mitigate MRSA risks in farming environments. Public health strategies should focus on prudent antimicrobial use and a One Health approach to curb the dissemination of antimicrobial resistance across humans, animals, and the environment.

**Supplementary Information:**

The online version contains supplementary material available at 10.1186/s40813-025-00459-0.

## Background

*Staphylococcus aureus (S. aureus)* is a bacterium found worldwide, known to colonise the nasal cavities of approximately 20% of humans, as well as a significant portion of mammalian and bird species, both wild and domestic [1–6]. While it primarily acts as a coloniser, the bacterium can also cause a broad range of clinical infections. These infections vary from minor skin and soft tissue infections to severe, life-threatening conditions such as sepsis, endocarditis, necrotising fasciitis, and pneumonia [7].

In its methicillin-susceptible form (MSSA), *S. aureus* can still cause significant morbidity and mortality, particularly in hospital settings [8–10]. However, the emergence of methicillin-resistant *Staphylococcus aureus* (MRSA) has exacerbated the challenges of managing infections caused by this bacterium. MRSA is of particular concern due to its resistance to commonly used β-lactam antibiotics, which are often the first line of treatment.

ß-lactam resistance in staphylococci primarily stems from two mechanisms. Enzymatic inactivation by β-lactamases encoded by the *blaZ* or *bla*_ARL_ genes or by target site replacement, mediated by the gene products of *mecA*,* mecB*, or *mecC*. Penicillin-resistant isolates possessing only *blaZ* are resistant to penicillinase-labile penicillins. In contrast, *S. aureus* isolates that are cefoxitin-resistant (MRSA) due to the presence of *mecA* are resistant to nearly all existing β-lactam antimicrobial agents, with the notable exception of ceftaroline, which lacks approval in veterinary medicine [11, 12]. MRSA can be transmitted through various pathways to humans, including direct human-to-human contact, environmental exposure, and the handling or consumption of contaminated milk or meat [8, 13, 14].

MRSA strains can be categorised into three groups based on their epidemiological and molecular characteristics: healthcare-associated MRSA (HA-MRSA), community-associated MRSA (CA-MRSA), and livestock-associated MRSA (LA-MRSA) [14–16]. Among these, LA-MRSA has gained attention as a zoonotic pathogen with the ability to transfer between animals and humans, particularly in intensive farming environments [7, 17–19]. The high potential for human colonisation following exposure to LA-MRSA has raised public health concerns [15, 20]. In hospital settings, it can be involved in nosocomial infections and can cause similar infections in humans, such as HA-MRSA or CA-MRSA [4, 7, 16, 21].

The frequency of LA-MRSA infections in hospitals has been linked to the density of livestock farms in the surrounding area [12, 21–24]. LA-MRSA is often detected in pig farms [25–28]. Among farmers and veterinarians, those who work directly with pigs are at the highest risk of LA-MRSA colonisation [29]. While direct contact with livestock is the primary risk factor for them acquiring LA-MRSA, inhalation of contaminated dust can increase the rate of LA-MRSA colonisation in exposed individuals [30, 31]. Clonal complex 398 (CC398) is the predominant MRSA lineage in pigs, ruminants, cats, dogs, and horses across Europe and in other regions globally [14, 17, 21, 26, 28, 32–35].

The biology of the house fly (HF) (*Musca domestica*) and stable fly (SF) (*Stomoxys calcitrans*) is closely linked to their roles as vectors of pathogens, particularly in environments like farm buildings, where both species are commonly found [36, 37]. Both the HF and SF have been described as potential vectors for viral and bacterial pathogens [38–41].

The HF is a highly adaptable insect that thrives in human habitats, particularly in areas where organic waste is abundant. Their life cycle is closely tied to environments that offer food and breeding grounds, such as garbage dumps, manure piles, and areas with decaying organic matter. The HF feeds on a wide variety of substrates, including animal and human excreta, decaying food, and other organic material [42]. Their proximity to humans and livestock, combined with their frequent contact with waste, animal or human excreta, or decaying substrates makes the HF a potential mechanical vector for the spread of infectious agents. They can carry pathogens on their body surfaces, in their saliva, and in their faeces. Studies have indicated that the guts of flies offer a conducive environment for harbouring antimicrobial-resistant bacteria and facilitating the horizontal transfer of plasmidic antimicrobial resistance genes in enterococci and *Escherichia coli (E. coli)* [43, 44].

The probability of mechanical transmission is heightened because the HF often moves between contaminated materials and food or surfaces used by humans [41, 45]. Studies have documented the presence of various pathogens, including MRSA, on the surface of the HF, highlighting their potential role in the spread of resistant bacteria [40, 45–47].

The SF, on the other hand, has a life cycle more directly associated with livestock. They are hematophagous, meaning they feed on the blood of animals, including cattle, pigs, horses, and occasionally humans. SF typically breed in decaying organic material, such as manure mixed with straw or other bedding material or wasted feed. Their larvae develop in these moist, organic environments, similar to the HF, but their adult feeding habits are more focused on blood meals [48].

Unlike the HF, which is primarily a nuisance pest, the SF inflicts direct harm on livestock through painful bites. Their feeding behaviour causes stress, discomfort, and even reduced productivity in animals due to blood loss and the energy expended to avoid or dislodge the flies [49].

Studies have detected viruses like African swine fever virus [50] and porcine circovirus 2 (PCV2) [39], but not porcine reproductive and respiratory syndrome virus (PRRSV) [38, 39], on the surface of SF collected in swine farms. Hemotropic mycoplasma, as well as *Staphylococcus* and enterobacterial species [39], have also been found. A recent case reported sows covered with bloody crusts across their bodies due to SF overpopulation; swab samples from these lesions tested positive for MRSA [51]. This finding demonstrates how SF infestations can contribute to MRSA transmission through skin wounds.

The role of dipteric flies as MRSA vectors is increasingly recognised; their presence, whether through biting (SF) or being drawn to pre-existing wounds (SF and HF), can exacerbate skin lesions in livestock and potentially facilitate MRSA infection.

### Aim of the study

The aim of this cross-sectional, descriptive study was to investigate the occurrence and characterisation of MRSA on HF and SF in Austrian piglet-producing farms. By isolating and characterising MRSA from the surface of flies, we intended to identify insights into possible transmission routes and potential impacts on the health of livestock and humans. We examined the molecular characteristics of isolated MRSA strains to gain insights into their origin, resistance mechanisms, and overall epidemiological significance.

## Methods

### Sample collection

**Fig. 1:**
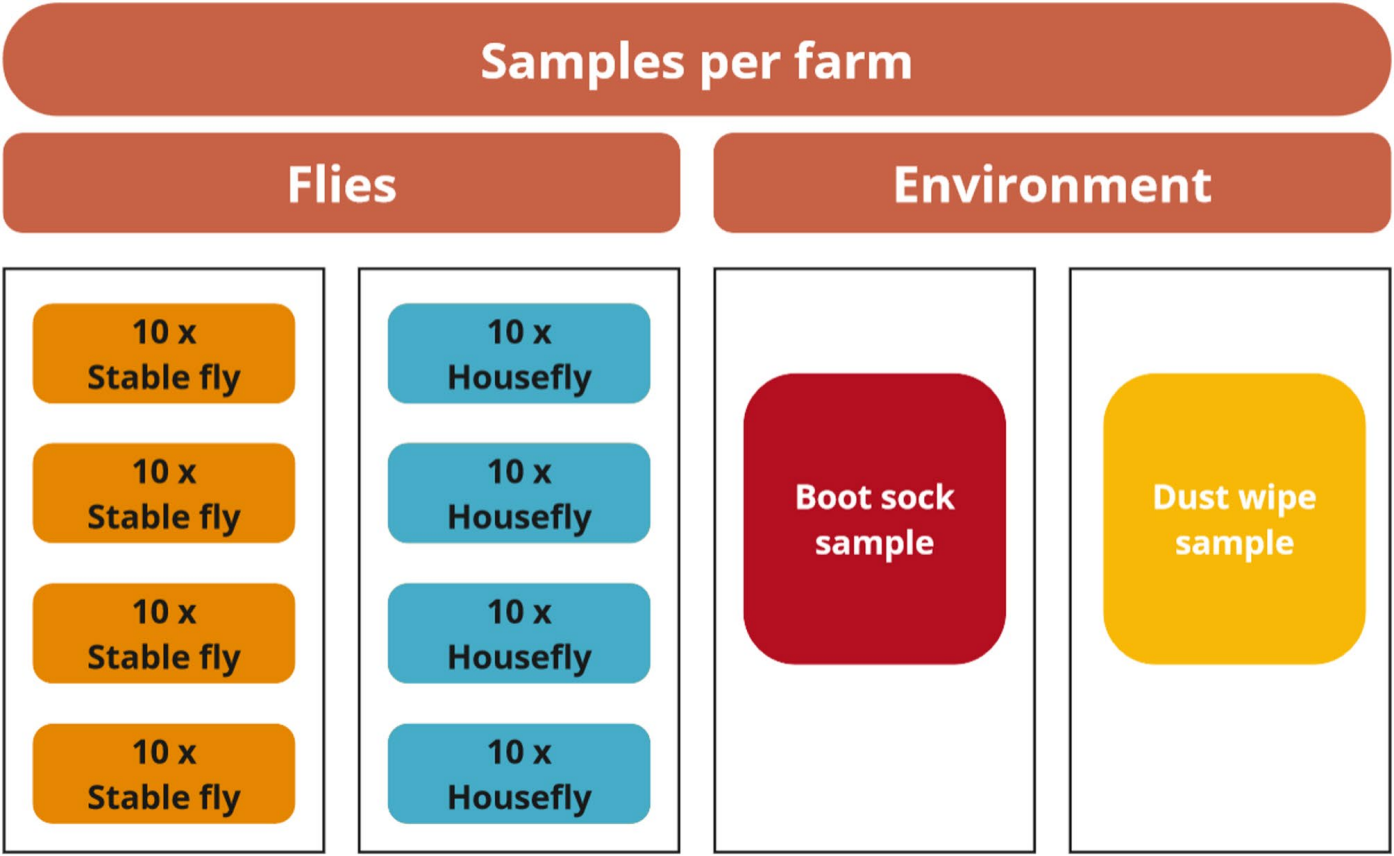
Sample collection setup. At each of the 24 piglet-producing farms, flies were collected using butterfly nets and pooled into four samples per species (house fly and stable fly), each consisting of 10 individuals. In addition, one boot sock sample (BSS) and one dust wipe sample (DWS) were collected per farm. This resulted in a total of 240 samples across all farms (96 HF, 96 SF, 24 BSS, 24 DWS). The same sampling procedure was applied consistently at each farm

**Fig. 2:**
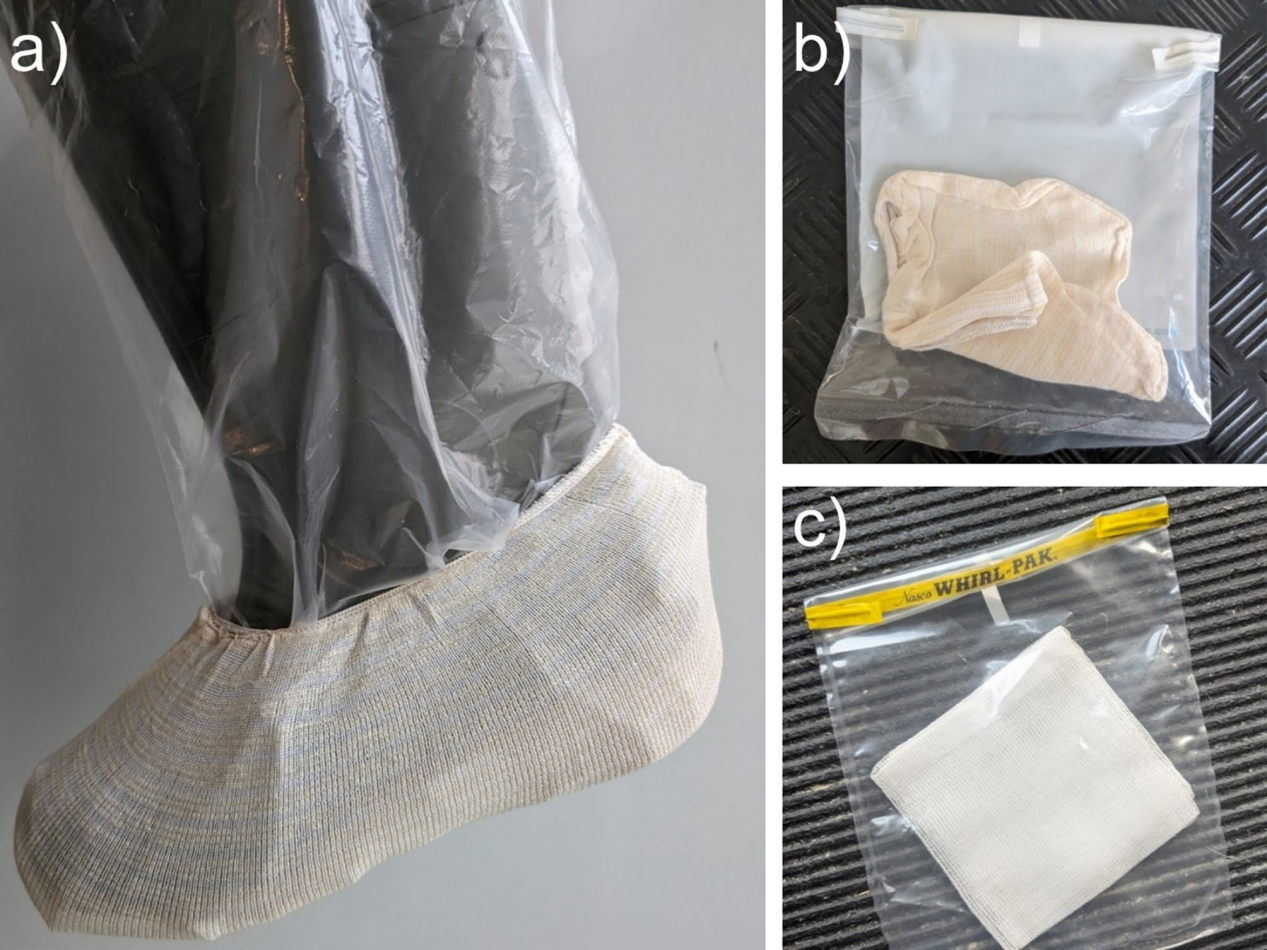
Sampling tools used for environmental MRSA detection. These images illustrate the standardised tools and techniques used for environmental sample collection across all farms. (**a**, **b**) Boot sock sample (BSS): A sterile disposable sock (SurfACE™, Romer Labs, Getzersdorf, Austria) was worn over a one-way use clean plastic boot cover and used to walk across the gestation area floor to collect surface debris and dust. (**c**) Dust wipe sample (DWS): Sterile gauze (HARTMANN, Heidenheim, Germany) was used to wipe surfaces such as stall equipment and windowsills wearing disposable one-way gloves

**Fig. 3:**
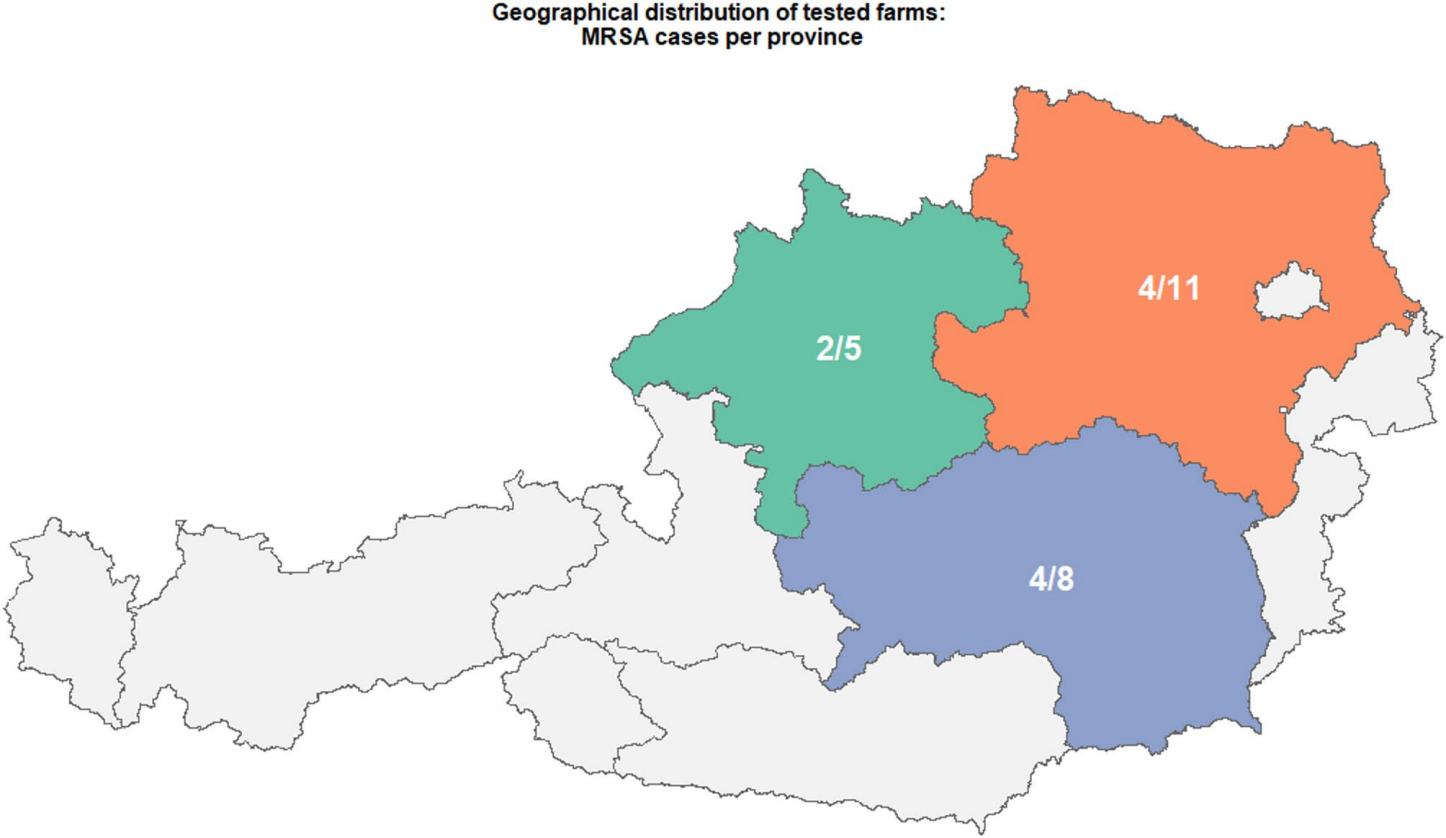
Province-level outcomes for tested farms. For each province, the X/Y label gives farms testing positive for MRSA (X) out of all farms sampled (Y): orange = Lower Austria, green = Upper Austria, blue = Styria

Samples were collected from 24 piglet-producing farms in Austria, each with at least 20 sows in production. Farms were selected for the study based primarily on whether both fly species were present simultaneously. Suitable farms were identified across three Austrian provinces: Lower Austria, Upper Austria, and Styria. We established contact either through the responsible veterinarian or directly via the farmers themselves. In some cases, farms were visited during the course of another ongoing project. After explaining the purpose of our study and obtaining permission from the farmers, we proceeded to collect the samples. The samples included HF and SF (four pooled samples of ten flies each), a boot sock sample (BSS), and a dust wipe sample (DWS) from each farm (Figs. 1 and 2). The distribution of farms across these provinces is illustrated in Fig. 3. Flies were collected using butterfly nets, with separate nets for each fly species to prevent cross-contamination of bacteria between the fly species. The nets used for fly collection were washed and disinfected with Gigasept^®^ (Schülke & Mayr GmbH, Norderstedt, Germany) after each use to avoid cross-contamination between farms. The flies were then transferred into sterile plastic tubes and kept under cooled conditions until further processing in the laboratory.

For the BSS, ready-to-use sterile SurfACE™ sampling devices (Romer Labs, Getzersdorf, Austria) were worn over plastic boot covers. Each DWS was collected by hand using sterile gauze wipes (HARTMANN, Heidenheim, Germany) and single-use gloves. The BSSs were taken from the floor of the barns in the gestation area, where fly specimens were collected. The DWSs were taken from surfaces such as stall equipment and windowsills in the gestation area.

### Fly washing and initial processing

Flies were washed within a 12-hour time window after sampling using a sterile physiological 0,9% saline solution (BRAUN™, Melsungen, Germany). One mL of solution was added to the flies, followed by thorough mixing on a vortexer for one minute at the highest speed. The washing solution was then transferred to a sterile tube for further analysis following standard microbiological research protocols [39].

### MRSA isolation and characterisation

For the isolation of MRSA, incubation in Tryptic Soy Broth with 6.5% (w/v) NaCl was performed at 37 °C for 24 to 48 h, followed by plating on a BBL™ CHROMagar™ MRSA II medium (Becton Dickinson (BD), Heidelberg, Germany). Colonies of *S. aureus* that displayed the distinctive colony morphology indicative of MRSA were selected for further characterisation. Species identification was additionally confirmed using Matrix-Assisted Laser Desorption/Ionisation-Time of Flight Mass Spectrometry (MALDI-TOF MS) (Microflex™ LRF MALDI-TOF mass spectrometer system; Bruker Daltonics, Bremen, Germany). Isolates were cryopreserved at -80 °C for further analysis [33, 52].

Antimicrobial susceptibility testing was performed using the agar disk diffusion method according to the standards of the Clinical and Laboratory Standards Institute (CLSI) [53]. The following antimicrobial agents were tested using the CLSI breakpoints for interpretation: cefoxitin (FOX 30 µg), ciprofloxacin (CIP 5 µg), gentamicin (GEN 10 µg), tetracycline (TET 30 µg), erythromycin (ERY 15 µg), clindamycin (CLI 2 µg), chloramphenicol (CHL 30 µg), trimethoprim-sulfamethoxazole (SXT 1.25/23.75 µg), nitrofurantoin (NIT 300 µg), rifampicin (RIF 5 µg) and linezolid (LZD 30 µg) (Becton Dickinson (BD), Heidelberg, Germany).

All isolates were genotyped using *spa* and *dru* typing as well as a DNA-based microarray analysis, as previously described by Loncaric et al. [34]. Isolate DNA extraction, whole-genome sequencing and genome assembly was performed as previously described [54]. For comparative genomics, whole-genome sequence (WGS)-based core genome multilocus sequence typing (cgMLST) analysis was performed using a scheme with 1,861 core genome targets followed by generation of minimum spanning trees (MST), all performed using the SeqSphere + software (Ridom, Münster, Germany) [34]. Information on classical multilocus sequence typing (MLST) was also extracted from the WGS data. In addition, the whole genome sequenced isolates were analysed with ABRicate (v1.1.0), CARD and ResFinder [55–59]. The sequencing data generated in this study have been deposited in the Sequence Read Archive (SRA) under BioProject accession number: PRJNA1289764.

### Data analysis

All data analyses were performed using RStudio [60]. Descriptive statistics were used to summarise the data. No formal hypothesis testing was conducted; therefore, no power calculation was performed. Visualisations were included to support interpretability and provide an accessible overview of the results.

## Results

**Fig. 4:**
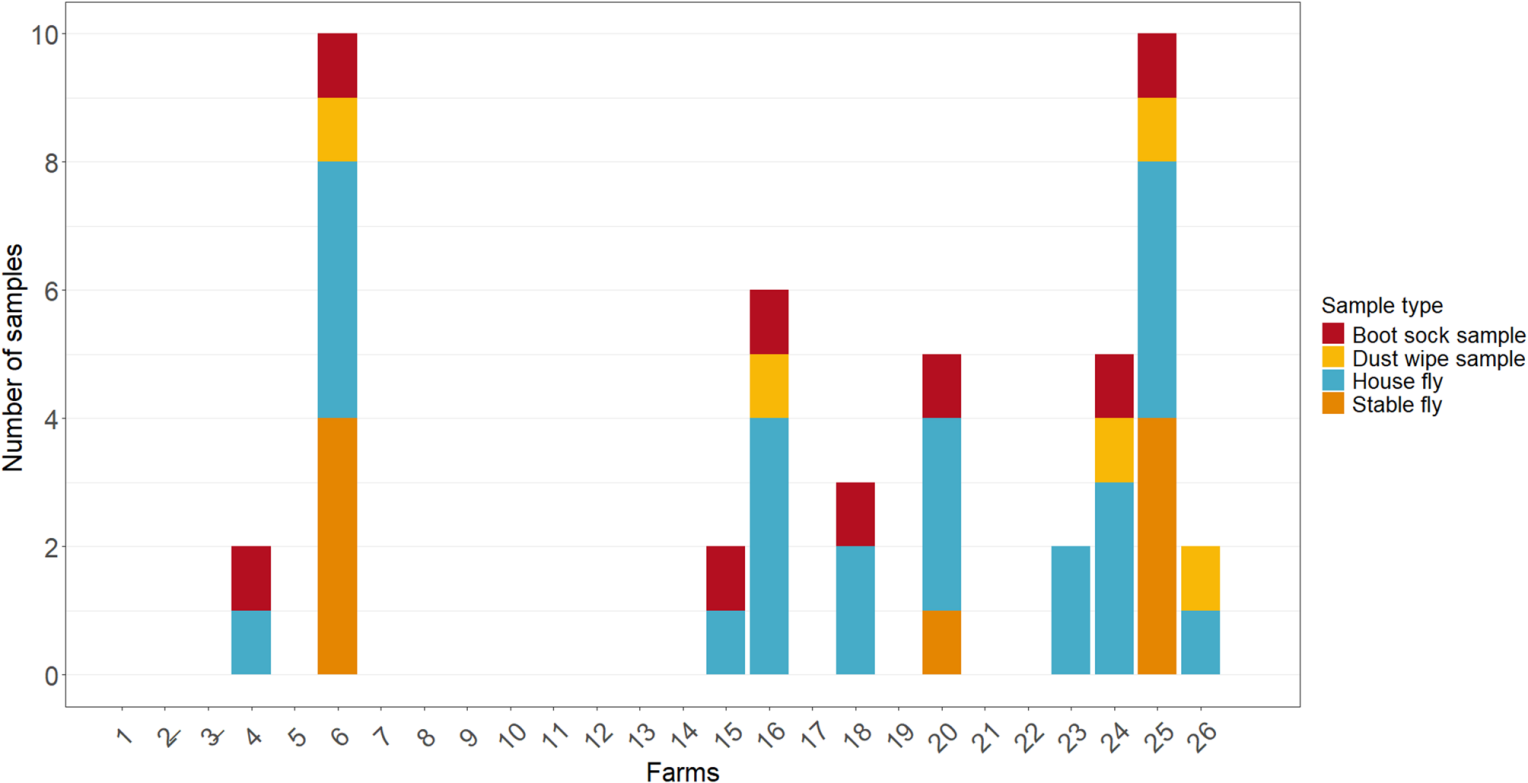
Stacked bar plot showing MRSA-positive samples per farm by sample type. Each bar represents one of the 24 investigated piglet-producing farms (Farms 2 and 3 excluded post-sampling). The total height of each bar corresponds to the number of MRSA-positive samples detected on that farm (maximum of 10 possible samples per farm). Each coloured segment within the bars indicates the sample type in which MRSA was detected. Farms with no positive samples are shown as bars with zero height

Of the 24 farms investigated, 10 (41.7%) had MRSA-positive samples. Of the 240 samples collected, MRSA was isolated from 47 samples, resulting in 47 isolates (19.6%). Two farms were excluded from the investigation, as the farmers withdrew their consent after sample collection. The number of positive samples varied widely among the farms (Fig. 4).

MRSA-positive results included 25 isolates from HFs (53.2% of all positives), 9 from SFs (19.1%), 8 from BSSs (17.0%), and 5 from DWSs (10.6%) (Table 1). The relative proportions of MRSA-positive samples across the sample categories show that the highest percentage of positive samples was found in BSSs (33.3%) and the lowest in SF samples (9.4%) (Table 1). All isolates displaying the distinctive colony morphology indicative of MRSA were confirmed as *S. aureus* and were resistant to cefoxitin and carried *mecA* gene. In addition to β-lactam resistance, resistance to tetracycline (100%), erythromycin (74%), and clindamycin (74%) was most frequently detected among the tested MRSA isolates. In contrast, resistance to ciprofloxacin (32%) and trimethoprim/sulfamethoxazole (17%) was less frequently observed (Table 1). This was reflected by the presence of the genes *mecA*, *blaZ*, *tet*(K), *tet*(M), *erm*(A), *erm*(B), *erm*(C), *lnu*(G), *lsa*(E), *vga*(E), *dfrG*, and in ciprofloxacin-resistant isolates, mutations in the quinolone resistance-determining regions (QRDRs) of the genes *gyrA* and *grlA* and were observed. In addition, spectinomycin resistant gene *spc*, streptomycin resistant genes *str*, and antiseptic resistant genes *qacC* and *qacG* were observed (Table 1, Additional file 1). All tested isolates were susceptible to linezolid, gentamicin, and rifampicin. Multidrug resistance (MDR), as defined by resistance to at least one agent in three different antimicrobial classes, was observed in 45 isolates [61].

**Table 1:** Summarized molecular characterization and antimicrobial resistance of the MRSA isolates investigated

Sample ID	Farm	Province*	Sample type**	spa	dru	ST***	CC§	SCCmec	cgMLST cluster	Phenotypic Resistance §§	Resistance genes	Amino acid alterations in QRDRs§§§	
26	4	LA	HF	t011	dt11a	ST398	CC398	V/VT	7	β-lactams, TET, ERY	*mecA*,* blaZ*,* blaI*,* blaR*,* tet*(K*)*,* tet*(M), *erm*(A), *qacC*,* qacG*		
32	4	LA	BSS	t011	dt6j	ST398	CC398	V/VT	none	β-lactams, TET, ERY	*mecA*,* blaZ*,* blaI*,* blaR*,* tet*(K), *tet*(M), *erm*(A), *qacG*		
42	6	LA	SF	t011	dt11a	ST398	CC398	V/VT	9	β-lactams, TET, ERY, CLI	*mecA*,* blaZ*,* blaI*,* blaR*,* tet*(K), *tet*(M), *erm*(B)		
46, 51	6	LA	SF	t011	dt11a	ST398	CC398	V/VT	10	β-lactams, TET, ERY, CLI	*mecA*,* blaZ*,* blaI*,* blaR*,* tet*(K), *tet*(M), *erm*(B), *qacC*		
43, 44, 45, 47, 48, 49, 50	6	LA	SF	t011	dt11a	ST10180	CC398	V/VT	1	β-lactams, TET, ERY, CLI	*mecA*,* blaZ*,* blaI*,* blaR*,* tet*(K), *tet*(M), *erm*(B)		
153	15	ST	HF	t011	dt11a	ST398	CC398	V/VT	7	β-lactams, TET	*mecA*,* blaZ*,* blaI*,* blaR*,* tet*(K), *tet*(M), *qacC*,* qacG*		
155	15	ST	BSS	t011	dt11a	ST398	CC398	V/VT	7	β-lactams, TET	*mecA*,* blaZ*,* blaI*,* blaR tet*(K), *tet*(M), *qacC*,* qacG*		
161	16	LA	HF	t1451	dt11df	ST398	CC398	V/VT	3	β-lactams, FQR, TET, CLI	*mecA*,* blaZ*,* blaI*,* blaR*,* tet*(K), *tet*(M), *lnu*(G), *dfrG*,* spc*,* qacG*	GrlA S80Y; GyrA S84L	
162	16	LA	HF	t034	dt11af	ST398	CC398	V/VT	3	β-lactams, FQR, TET	*mecA*,* blaZ*,* blaI*,* blaR*,* tet*(K), *tet*(M), *dfrG*	GrlA S80Y; GyrA S84L	
163	16	LA	HF	t011	dt11af	ST398	CC398	V/VT	3	β-lactams, FQR, TET, CLI	*mecA*,* blaZ*,* blaI*,* blaR*,* tet*(K), *tet*(M), *lnu*(G), *dfrG*,* spc*	GrlA S80Y; GyrA S84L	
164	16	LA	HF	t034	5a-2d-4a-0-2d-6f-2a-2 g-3b-4e-3e	ST398	CC398	V/VT	3	β-lactams, FQR, TET, CLI	*mecA*,* blaZ*,* blaI*,* blaR*,* tet*(K), *tet*(M), *lnu*(G), *dfrG*,* spc*	GrlA S80Y; GyrA S84L	
165	16	LA	BSS	t034	dt11af	ST398	CC398	V/VT	3	β-lactams, FQR, TET	*mecA*,* blaZ*,* blaI*,* blaR*,* tet*(K), *tet*(M), *dfrG*	GrlA S80Y; GyrA S84L	
166	16	LA	DSF	t034	dt11af	ST398	CC398	V/VT	3	β-lactams, FQR, TET	*mecA*,* blaZ*,* blaI*,* blaR*,* tet*(K), *tet*(M), *dfrG*	GrlA S80Y; GyrA S84L	
181	18	UA	HF	t011	dt11a	ST398	CC398	V/VT	5	β-lactams, FQR, TET, ERY	*mecA*,* blaZ*,* blaI*,* blaR*,* tet*(K), *tet*(M)		
182	18	UA	HF	t011	dt11a	ST398	CC398	V/VT	5	β-lactams, FQR, TET	*mecA*,* blaZ*,* blaI*,* blaR*,* tet*(K), *tet*(M)		
185	18	UA	BSS	t011	dt11a	ST398	CC398	V/VT	5	β-lactams, FQR, TET, ERY	*mecA*,* blaZ*,* blaI*,* blaR*,* tet*(K), *tet*(M)		
200	20	ST	SF	t011	dt11v	ST398	CC398	V/VT	8	β-lactams, TET, ERY, CLI	*mecA*,* blaZ*,* blaI*,* blaR*,* tet*(K), *tet*(M), *erm*(B), *lnu*(G), *lsa*(E), *dfrG*,* spc*,* qacG*		
202	20	ST	HF	t034	5a-2d-4a-2d-2d-6f-3a-2 g-3b-4e-3e	ST398	CC398	V/VT	none	β-lactams, TET, CLI	*mecA*,* blaZ*,* blaI*,* blaR*,* tet*(K), *tet*(M), *erm*(B), *lnu*(G), *lsa*(E), *dfrG*,* spc*		
203	20	ST	HF	t034	5a-2d-4a-2d-2d-6f-3a-2 g-3b-4e-3 g	ST398	CC398	V/VT	none	β-lactams, TET, CLI	*mecA*,* blaZ*,* blaI*,* blaR*,* tet*(K), *tet*(M), *erm*(B), *lnu*(G), *lsa*(E), *dfrG*,* spc*		
204	20	ST	HF	t011	dt11v	ST398	CC398	V/VT	8	β-lactams, TET, ERY, CLI	*mecA*,* blaZ*,* blaI*,* blaR*,* tet*(K), *tet*(M), *erm*(B), *lnu*(G), *lsa*(E), *dfrG*,* spc*		
205	20	ST	BSS	t034	dt11a	ST398	CC398	V/VT	none	β-lactams, TET, CLI	*mecA*,* blaZ*,* blaI*,* blaR*,* tet*(K), *tet*(M), *lnu*(G), *lsa*(E), *dfrG*,* spc*,* str*		
233	22	LA	HF	t010	dt11a	ST397	CC397	V/VT	none	β-lactams, FQR, TET, ERY	*mecA*,* blaZ*,* blaI*,* blaR*,* tet*(K), *tet*(M), *str*		
234	23	LA	HF	t011	dt11a	ST398	CC398	V/VT	10	β-lactams, FQR, TET, ERY	*mecA*,* blaZ*,* blaI*,* blaR*,* tet*(K), *tet*(M), *str*		
241, 243, 244	24	UA	HF	t034	dt11ax	ST398	CC398	V/VT	4	β-lactams, TET, ERY, CLI, SXT	*mecA*,* blaZ*,* blaI*,* blaR*,* tet*(K), *tet*(M), *erm*(C), *lnu*(G), *lsa*(E), *dfrG*,* spc*,* qacC*,* qacG*		
245	24	UA	BSS	t034	dt11ax	ST398	CC398	V/VT	4	β-lactams, TET, ERY, CLI, SXT	*mecA*,* blaZ*,* blaI*,* blaR*,* tet*(K), *tet*(M), *erm*(C), *lnu*(G), *lsa*(E), *dfrG*,* spc*,		
246	24	UA	DSF	t571	dt11ax	ST398	CC398	V/VT	4	β-lactams, TET, ERY, CLI, SXT	*mecA*,* blaZ*,* blaI*,* blaR*,* tet*(K), *tet*(M), *erm*(C), *lnu*(G), *lsa*(E), *dfrG*,* spc*,* qacC*,* qacG*		
247	25	ST	SF	t571	dt5e	ST398		V/VT	6	β-lactams, FQR, TET, ERY, CLI	*mecA*,* blaZ*,* blaI*,* blaR*,* tet*(K), *tet*(M), *erm*(A), *vga*(E), *dfrG*,* spc*	GrlA S80F; GyrA S84L	
248	25	ST	SF	t011	dt5e	ST398	CC398	V/VT	2	β-lactams, FQR, TET, ERY, CLI	*mecA*,* blaZ*,* blaI*,* blaR*,* tet*(K), *tet*(M), *erm*(A), *vga*(E), *dfrG*,* spc*		
249	25	ST	SF	t571	dt5e	ST398	CC398	V/VT	2	β-lactams, TET, ERY, CLI	*mecA*,* blaZ*,* blaI*,* blaR*,* tet*(K), *tet*(M), *erm*(A), *vga*(E), *dfrG*,* spc*		
250	25	ST	SF	t011	dt5e	ST398	CC398	V/VT	2	β-lactams, TET, ERY, CLI, SXT	*mecA*,* blaZ*,* blaI*,* blaR*,* tet*(K), *tet*(M), *erm*(A), *vga*(E), *dfrG*,* spc*		
251, 252, 253, 254	25	ST	HF	t034	dt5e	ST398	CC398	V/VT	2	β-lactams, TET, ERY, CLI	*mecA*,* blaZ*,* blaI*,* blaR*,* tet*(K), *tet*(M), *erm*(A), *vga*(E), *dfrG*,* spc*		
255	25	ST	BSS	t011	dt5e	ST398	CC398	V/VT	6	β-lactams, FQR, TET, ERY, CLI, SXT	*mecA*,* blaZ*,* blaI*,* blaR*,* tet*(K), *tet*(M), *erm*(A), *vga*(E), *dfrG*,* spc*	GrlA S80F; GyrA S84L	
256	25	ST	DSF	t011	dt5e	ST398	CC398	V/VT	6	β-lactams, FQR, TET, ERY, CLI, SXT	*mecA*,* blaZ*,* blaI*,* blaR*,* tet*(K), *tet*(M), *erm*(A), *vga*(E), *dfrG*,* spc*	GrlA S80F; GyrA S84L	
263	26	ST	HF	t011	dt11a	ST398	CC398	V/VT	10	β-lactams, TET, ERY, CLI	*mecA*,* blaZ*,* blaI*,* blaR*,* tet*(K), *tet*(M), *str*		
266	26	ST	DSF	t1451	dt11a	ST398	CC398	[V/VT + ccrA(B)1]	9	β-lactams, TET, ERY, CLI	*mecA*,* blaZ*,* blaI*,* blaR*,* tet*(K), *tet*(M)		
*Province: LA: Lower Austria, ST: Styria, UA: Upper Austria													
** HS: house fly, BSS: boot sock sample, SF: stable fly, DWS: dust wipe sample													
*** ST: sequence type													
§ CC: clonal complex													
§§ FQR: fluoroquinolone resistant, TET: tetracycline, ERY: erythromycin, CLI: clindamycin, SXT: trimethoprim-sulfamethoxazole													
§§§ QDRD: quinolone resistance-determining regions													

**Fig. 5:**
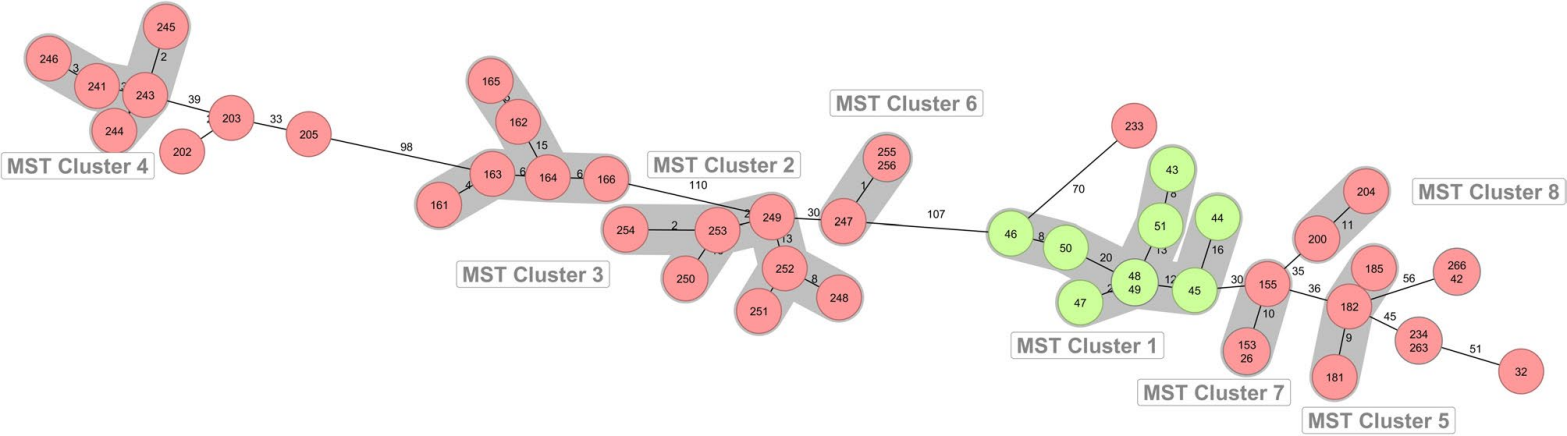
Minimum spanning tree for MRSA isolates based on the cgMLST of S. aureus. Colors correspond to the sequence types (salmon = 398, green = unknown). Each circle represents one or more isolates. Numbers between circles refer to the allelic differences between two isolates. Closely related isolates were identified with a maximum of 24 allelic differences and are shaded in grey

All identified MRSA strains belonged to the clonal complex CC398, with 38 belonging to sequence type (ST) 398 whereas remaining nine strains belong to new ST10180. All isolates belong to SCC*mec* type V. Four different *spa* types t011, t034, t1451, or t571, and seven *dru* types dt11a, dt11af, dt11ax, dt11df, dt11v, dt5e dt6j with *spa* type t011 and *dru* type dt11a being predominant. Three strains could not be assigned to known *dru* type (Table 1). For comparative genomics, a whole-genome-based cgMLST phylogenetic analysis was conducted on all isolates, and a minimum spanning tree (MST) was generated (Fig. 5). Allelic distances between isolates ranged from zero to a maximum of 110. Applying a defined cluster threshold (CT) of 24 allelic differences resulted in the identification of eight distinct clusters (Fig. 5). Study results (including sample types, phenotypic, and genomic characteristics of the isolates) are summarized in Table 1 and/or are available in the supplement as Additional File 1.

## Discussion

The study investigated prevalence and characteristics of LA-MRSA in Austrian pig farms. The selection of farms from Lower Austria, Upper Austria, and Styria aligns with the regional distribution of pig farming in Austria. As of June 1, 2024, these three provinces collectively accounted for 94.0% of the Austrian pig population, underscoring their prominence in the country’s swine industry [62]. This concentration is attributed to favourable agricultural conditions, particularly the availability of arable land suitable for swine feed production. All examined isolates in this study belonged to CC398, a clonal complex closely associated with livestock farming, particularly in pigs. The findings align with several European studies that reported a high prevalence of MRSA in pig populations, also identifying ST398 as the dominant [26, 63–66].

Most isolates in this study carried SCC*mec* type V, commonly associated with CC398. Compared to the CC398 from Austrian companion animals where SCC*mec* IV is predominant, the SCC*mec* V type is reported less often [33, 34]. The antimicrobial resistance profile observed aligns with patterns frequently reported for livestock-associated CC398 SCC*mec* V isolates. Tetracycline resistance, consistently linked to the *tet*(M) gene, often in combination with *tet*(K), as in this study, or *tet*(L), are characteristic of this clonal complex [67–69]. Phenotypic resistance to macrolides and lincosamides correlates with the presence of *erm*(A), *erm*(C), *lnu*(G), *lsa*(E), *vga*(E), which is in agreement with findings from other studies [66, 70, 71]. Fluoroquinolone resistance in *S. aureus* is commonly associated with point mutations in the quinolone resistance-determining regions (of the *gyrA*, *gyrB*, *grlA*,* grlB* and *norA* genes [66, 72–74]. In the present study, point mutations mediating fluoroquinolone resistance were detected in *grlA* and *gyrA* genes of ciprofloxacin-resistant isolates. These mutations resulted in the amino acid exchanges S80Y and S84L, respectively, which are previously observed in CC398 strains of porcine origin [66]. In addition, non-ß-lactam resistance genes such as trimethoprim resistant gene *dfrG*, spectinomycin resistant gene *spc*, streptomycin resistant genes *str* are frequently associated with porcine CC398 MRSA strains [66]. The presence of *qacC and qacG* genes, which encode the quaternary ammonium compound (QAC), suggests potential tolerance to disinfectants. While biocide resistance testing was not conducted in this study, these findings highlight the need for biocide susceptibility testing alongside antimicrobial testing, as biocides may contribute to resistance selection. Studies on the prevalence of *qac* genes in pig farming environments reflect this need. In MRSA CC30 isolates from Danish pigs, *qacG* and *qacC* genes were identified, providing early evidence of QAC resistance as well as in Germany in MRSA CC398 [66, 75]. Additionally, metagenomic analyses have revealed the co-occurrence of biocide, antibiotic, and metal resistance genes in pig microbiomes, which may contribute to the proliferation of antimicrobial resistance in pig farming settings [76] Beyond biocide resistance, examining virulence gene diversity provides insights into the evolution of bacterial pathogenicity. Studying bacteria from livestock, particularly in pig farming, contributes to understanding the interplay between animal health, farming practices, and environmental factors, aligning with the One Health approach.

In some instances, MRSA strains associated with flies belonged to the same cgMLST cluster also found in environmental samples (BSS, DWS) from the same farm, suggesting that these insects may transmit MRSA in and around the farms. This suggests that clonal expansion and within-farm transmission may play a key role in the persistence of specific MRSA types However, not all strains overlapped perfectly between flies and other environmental samples, indicating that flies may acquire MRSA from multiple sources. It remains unknown whether MRSA persists primarily in or on flies or in barn dust, especially during the intervals between piglet production cycles. Future longitudinal and interventional studies, such as sampling both flies and the barn environment over time, before and after cleaning, could clarify whether flies serve as short-term mechanical vectors or contribute to long-term MRSA persistence. On the other hand, in some cases, we observed the same cgMLST cluster among MRSA isolates originating from different farms, but also different cgMLST clusters within the same farm. This strongly suggests a clonal distribution of CC398 MRSA between Austrian farms.

The findings of the present study have significant public health implications. Farmers and veterinarians working in close proximity to pigs are at a higher risk of LA-MRSA colonisation [19]. Previous studies have shown a nasal colonisation rate of 13% among Austrian veterinarians, particularly those with more than three farm visits per week [18]. Additionally, exposure to farm dust, which can carry MRSA, poses a risk not only within the farm environment but also when transported to other locations [31]. Notably, there is a strong correlation between nasal carriage of MRSA and the number of working hours in barns, with barn air being a significant factor for exposure to ST398 MRSA [30].

In our study, BSSs were positive in 8/11 MRSA-positive farms, and DWSs were positive in 5/11 cases, confirming the widespread presence of MRSA in the barn environment. These findings highlight the need for stringent biosecurity protocols and preventive measures, such as wearing gloves, masks, and practicing regular hygiene to reduce the risk of MRSA colonisation and transmission.

The role of flies as vectors of MRSA transmission is increasingly being recognised. In our study, LA-MRSA was detected in both the HF and SF, supporting previous findings by Stelder and colleagues [40] that the HF is more likely to carry MRSA. By sampling the two fly species separately, we minimised cross-contamination, suggesting a true carriage of MRSA by both of these dipteric flies. This adds to the growing body of evidence linking flies to the transmission of MRSA in farming environments [26].

One hypothesis is that flies killed by adulticides might be inadvertently consumed by pigs, thereby contributing to bacterial transmission. This underscores the need for comprehensive insect control, which should address both adult flies and larval development sites, and properly managing dead insects. Effective measures include initiating fly control immediately after cleaning gestation areas to prevent buildup, regularly emptying slurry channels to remove breeding sites, and installing non-slatted plates around feeding areas to limit feed spillage. Rapid intervention with adulticides and larvicides in high-density fly areas is essential to maintaining animal health [39, 42, 77].

Constant and consistent monitoring of the fly population is critical for early intervention, particularly for the SF, which can cause skin lesions in pigs. If these lesions become colonised by staphylococci and other bacteria, persistent infection may occur unless both the SF population and wound sites are properly managed. To enhance animal health and farm performance, pest insect control should be combined with local wound treatment using zinc or silver sprays [51].

Prior to this study, investigations on LA-MRSA in Austrian swine farms were limited. The baseline study by the European Food Safety Authority (EFSA) in 2008 reported a 12.6% prevalence in various Austrian pig farms, all attributed to ST398 [78]. Our findings provide new insights into LA-MRSA epidemiology in swine farms, suggesting that the flies could act as living mechanical vectors. The detection of MRSA in washing solutions (potentially containing fly excreta and saliva) indicates the possibility of both external and internal colonisation. Still, additional factors, such as antibiotic usage and other entry routes, must be addressed. A comprehensive approach is needed to protect animal and public health that combines targeted fly control with a broader antimicrobial stewardship approach.

Despite providing new insights into LA-MRSA prevalence and resistance profiles in Austrian pig farms, this study has several limitations. The relatively small sample size and the withdrawal of two farms limit the generalizability of our findings. The cross-sectional design captures MRSA prevalence at a single point in time, making it difficult to draw conclusions about potential seasonal or longitudinal fluctuations. Finally, the lack of detailed antibiotic usage data and risk factor analysis highlights the need for more comprehensive research to fully grasp the drivers of LA-MRSA spread and persistence in Austrian pig farming. Therefore, further studies are necessary to understand the role of MRSA in dipteric flies and in the broader farm environment, ultimately clarifying how MRSA persists and spreads within pig populations.

## Conclusion

This study confirmed the presence of MRSA in Austrian pig farms and identified HF and SF as potential vectors. Their potential role points to the broader challenge of antimicrobial resistance in livestock settings rather than serving as the primary problem. Environmental contamination, as evidenced by both BSS and DWS, emphasises the critical need for stringent hygiene, thorough sanitisation, and robust biosecurity measures.

From a public health perspective, responsible antimicrobial use in livestock production is vital to mitigating resistance development and its transfer to humans. Overall, the findings underscore the importance of a One Health approach, integrating human, animal, and environmental health strategies to address and effectively contain MRSA spread.

## Supplementary Information

Below is the link to the electronic supplementary material.

Supplementary Material 1

## Data Availability

No datasets were generated or analysed during the current study.

## Abbreviations

MSSA: Methicillin-susceptible *Staphylococcus aureus*
MRSA: Methicillin-resistant *Staphylococcus aureus*
mecA, mecB, mecC: Genes conferring methicillin resistance in *Staphylococcus aureus*
QRDR: quinolone resistance-determining region
HA-MRSA: Healthcare-associated methicillin-resistant *Staphylococcus aureus*
CA-MRSA: Community-associated methicillin-resistant *Staphylococcus aureus*
LA-MRSA: Livestock-associated methicillin-resistant *Staphylococcus aureus*
EFSA: European Food Safety Authority
ECDC: European Centre for Disease Prevention and Control
CC398: Clonal complex 398
ASF: African swine fever
PCV2: Porcine circovirus 2
PRRSV: Porcine reproductive and respiratory syndrome virus
PVL: Panton-Valentine Leukocidin
BSS: Boot sock sample
DWS: Dust wipe sample
TSB: Tryptic Soy Broth
CLSI: Clinical and Laboratory Standards Institute
FOX: Cefoxitin
CIP: Ciprofloxacin
GM: Gentamicin
TET: Tetracycline
ERY: Erythromycin
CLI: Clindamycin
CHL: Chloramphenicol
SXT: Trimethoprim-sulfamethoxazole
NIT: Nitrofurantoin
RIF: Rifampicin
LZD: Linezolid
WGS: whole-genome sequencing
cgMLST: core genome multilocus sequence typing
MST: minimum spanning trees
MDR: Multidrug resistance
spa: Staphylococcal protein A (typing)
dru: Direct repeat units (typing)
QAC: quaternary ammonium compound

## Declarations

### AI-Assisted Copy-Editing Statement

In accordance with the publisher’s guidelines, we used ChatGPT solely for AI-assisted copy editing, which included refining grammar, spelling, punctuation, and style. The authors confirm that no generative editorial work or autonomous content creation was performed by the AI, and that final responsibility for the manuscript’s content remains with the authors.
